# Risk Factors and Reasons for Treatment Abandonment for Patients With Esophageal Atresia: A Study From a Tertiary Care Hospital in Beijing, China

**DOI:** 10.3389/fped.2021.634573

**Published:** 2021-04-27

**Authors:** Shen Yang, Junmin Liao, Siqi Li, Kaiyun Hua, Peize Wang, Yanan Zhang, Yong Zhao, Yichao Gu, Shuangshuang Li, Jinshi Huang

**Affiliations:** Department of Neonatal Surgery, National Center for Children's Health, Beijing Children's Hospital, Capital Medical University, Beijing, China

**Keywords:** treatment abandonment, esophageal atresia, risk factors, reasons, children

## Abstract

**Background:** This study aims to identify the risk factors and reasons for treatment abandonment for patients with esophageal atresia (EA) in a tertiary care hospital in China.

**Methods:** A retrospective study was conducted on 360 patients with EA admitted to Beijing Children's Hospital between January 1, 2007 and June 1, 2020. Medical records for treatment abandonment and non-treatment abandonment patients were compared. Univariate and multivariate logistic regression analyses were conducted to identify potential risk factors for treatment abandonment.

**Results:** After the diagnosis of EA, parents of 107 patients refused surgical repair and discharged against medical advice, and 253 patients underwent surgical repair. Among these 253 patients, parents of 59 patients abandoned treatment after surgery; 52 patients were discharged in an unstable condition, and parents of seven patients abandoned resuscitation leading to death in the hospital. By comparing clinical characteristics between treatment abandonment before surgery (*n* = 107) and non-treatment abandonment (*n* = 253) groups, we found that mother's parity >1, unplanned admission to intensive care unit before surgery, associated anomalies, and Gross type A/B were significant independent risk factors for treatment abandonment before surgery. Furthermore, birth weight <2,545 g, being discharged from neonatal center/intensive care unit and other departments, unplanned admission to intensive care unit after surgery, operative time >133 min, admission before 2016, pneumothorax, and anastomotic leakage were significant independent risk factors for treatment abandonment after surgery. The reasons for treatment abandonment included financial difficulties, multiple malformations with poor prognosis, belief of incurability and concerns about the prognosis of the diseases, postoperative complications, and extensive length of intensive care unit stay.

**Conclusions:** Treatment abandonment of children with EA/TEF is still a common and serious problem in China. This study showed that EA/TEF patients in critical conditions, with associated anomalies, Gross type A/B, and who had occurrence of complications had high-risk for treatment abandonment.

## Introduction

Esophageal atresia (EA) and tracheoesophageal fistula (TEF) is one of the most common congenital malformations of the esophagus, with an incidence of 1/2,500–1/4,500 (1). The survival rate of EA/TEF without severe malformation reported in the relevant literature is higher than 90% (1). The prognosis for patients with EA/TEF has greatly improved with advances in surgical techniques and preoperative and postoperative care. However, short-term complications after EA/TEF repair include anastomotic leakage, anastomotic stricture, and recurrent TEF (2). Furthermore, EA/TEF is also associated with numerous long-term comorbidities that affect the esophagus and respiratory system (1).

In developing countries including China, some parents of newborns with EA/TEF will choose to abandon treatment before or after the surgery. Possible reasons include the critical condition of the newborn, the combination of multiple malformations, family socioeconomic factors, and concerns about the prognosis of the disease. There is no doubt that treatment abandonment may lead to the worsening or death of patients and increase the chance of readmission.

In developed countries, any refusal or abandonment is likely to lead to health and social services intervening and court action might be taken to ensure that the child receives treatment. Unfortunately, such state support and intervention does not exist in China, and treatment refusal and abandonment remain common events. Treatment abandonment for children with EA/TEF is not merely a simple medical problem, but a complex challenge involving ethics, health economics, sociology and other fields. Many studies focus on the abandonment of treatment for children's cancer and analyze the risk factors (3–5). However, few studies focus on treatment abandonment for EA/TEF in developing countries. This study aims to explore the relevant factors and reasons of treatment abandonment by reviewing medical records in our center.

## Methods

### Patients and Clinical Characteristics

We retrospectively collected the medical records of all patients with EA admitted to the Beijing Children's Hospital between January 1, 2007 and June 1, 2020. Their demographic information, preoperative assessments, operative details, and surgical complications were extracted from the electronic medical records and analyzed. Treatment abandonment was defined as abandonment before surgical repair of EA, or discharge against medical advice after surgery in an unstable condition (including refusal of resuscitation, impromptu removal of tracheal intubation, thoracic drainage, gastric tube, etc.), and signing of a treatment abandonment document (decision of parents to stop medical treatment for their child after discussion with medical staff). All methods were carried out in accordance with relevant guidelines and regulations, and the study was approved by the Medical Ethics Committee of Beijing Children's Hospital (2019-k-333). A waiver of consent was provided for the analyses conducted in this study.

### Statistical Analysis

Statistical analysis was performed by SPSS 22.0. Continuous variables were presented as the mean and standard deviation for normal distribution or median and interquartile range for non-normal distribution. Categorical variables were reported as counts and percentages. Two independent sample *t* tests and χ^2^ tests were used to compare characteristics between the treatment abandonment and non-treatment groups. Receiver operating characteristic (ROC) curve analysis was performed to determine the most appropriate cutoff values. Univariate and multivariate logistic regression analyses were conducted to select potentially useful characteristics for predicting treatment abandonment. *P* < 0.05 was considered statistically significant.

## Results

### Patient Characteristics

In this study, 360 patients were included in the analysis (226 boys and 134 girls). These patients had a median gestational age of 39 weeks (range: 32 to 44 weeks) and a median birth weight of 2,925 g (range: 1,500–4,500 g). Many patients were found to have other congenital diseases, including non-syndromic anomalies (*n* = 131), VACTERL syndrome (*n* = 33), chromosome abnormality (*n* = 3), syndromic diagnosis (*n* = 1), none anomalies (*n* = 153), and unknown (*n* = 39). According to the Gross classification, the types of initial EA/TEF were type A (*n* = 12), type B (*n* = 1), type C (*n* = 267), type D (*n* = 10), type E (*n* = 48), and unknown (*n* = 22).

After the diagnosis of EA/TEF, 253 patients underwent surgical repair and parents of 107 patients refused surgical repair and discharged against medical advice. The primary operations were performed *via* thoracoscopic (*n* = 68, including six operations that were converted to an open thoracotomy) or open (*n* = 177) approaches. In addition, eight patients only underwent exploratory surgery or gastrostomy and did not obtain esophageal continuity. Fifty-two patients were in an unstable condition after surgery when they were discharged against medical advice, and seven patients who refused resuscitation died in the hospital. Among the other 194 patients, after a median follow-up of 83 months (range: 5–160 months), 160 patients survived, six died, and 28 were lost to follow-up.

### Comparison Between Treatment Abandonment Before Surgery and Non-treatment Abandonment Groups

As shown in Table 1, by comparing clinical characteristics between treatment abandonment before surgery (*n* = 107) and non-treatment abandonment (*n* = 253) groups, we found significant differences in age at diagnosis, mother's gravidity and parity, existence of twin, mother's occupation, existence of a sibling, existence of an elder brother, urgency of admission, department of admission, department of discharge, unplanned admission to intensive care unit before surgery, Gross classification, associated anomalies, duration of hospital stay, duration of stay in intensive care unit, and hospitalization expense (all *P* < 0.05). However, there were no differences in other characteristics between the two groups (all *P* > 0.05).

**Table 1:** Comparison between treatment abandonment before surgery and non-treatment abandonment groups.

**Variables**	**Treatment abandonment before surgery (*n* = 107)**	**Non-treatment abandonment (*n* = 253)**	**Results**	***P*-value**
Gender (*n*, %)			0.269	0.604
Boy	65 (60.7)	161 (63.6)		
Girl	42 (39.3)	92 (36.4)		
Age at diagnosis (median, days)	1 (1, 3)	2 (1, 7)	−4.004	<0.001
Birth weight (mean, g)	2,850 ± 540	2,924 ± 493	−1.267	0.206
Gestational age (*n*, %)			1.560	0.212
Preterm	15 (14.7)	25 (10.0)		
Term/overdue	87 (85.3)	224 (90.0)		
Mode of delivery (*n*, %)			2.346	0.126
Cesarean delivery	57 (55.9)	159 (64.6)		
Spontaneous vaginal delivery	45 (44.1)	87 (35.4)		
Gravidity (median)	2.0 (1.0, 3.0)	2.0 (1.0, 3.0)	−2.739	0.006
Parity (median)	2.0 (1.0, 2.0)	2.0 (1.0, 2.0)	−2.740	0.006
Twin (*n*, %)			5.346	0.008
No	100 (100.0)	233 (93.6)		
Yes	0	16 (6.4)		
Geographic location (*n*, %)			2.828	0.093
City	45 (42.5)	131 (52.2)		
Village	61 (57.5)	120 (47.8)		
Father's occupation (*n*, %)			4.295	0.117
Office staff/vendor/worker	34 (55.7)	125 (70.2)		
Farmer	17 (27.9)	34 (19.1)		
Unemployed	10 (16.4)	19 (10.7)		
Mother's occupation (*n*, %)			7.632	0.022
Office staff/vendor/worker	17 (31.5)	88 (51.8)		
Farmer	18 (33.3)	33 (19.4)		
Unemployed	19 (35.2)	49 (28.8)		
Father's age (median, years)	30 (26, 34)	30 (27, 35)	−0.434	0.664
Mother's age (median, years)	28 (25, 33)	29 (26, 33)	−0.405	0.686
Siblings (*n*, %)			8.477	0.004
Yes	56 (54.4)	93 (37.5)		
No	47 (45.6)	155 (62.5)		
Elder brother (*n*, %)			10.553	0.001
Yes	26 (27.7)	28 (12.6)		
No	68 (72.3)	194 (87.4)		
Elder sister (*n*, %)			2.329	0.127
Yes	25 (26.6)	42 (18.9)		
No	69 (73.4)	180 (81.1)		
Urgency of admission (*n*, %)			11.540	0.001
Emergency	92 (86.0)	174 (68.8)		
Planned	15 (14.0)	79 (31.2)		
Department of admission (*n*, %)			25.975	<0.001
Neonatal surgery	46 (43.0)	168 (66.4)		
Neonatal center/intensive care unit	58 (54.2)	67 (26.5)		
Others	3 (2.8)	18 (7.1)		
Department of discharge (*n*, %)			12.678	0.002
Neonatal surgery	43 (40.2)	142 (56.1)		
Neonatal center/intensive care unit	61 (57.0)	94 (37.2)		
Others	3 (2.8)	17 (6.7)		
Unplanned admission to intensive care unit before surgery (*n*, %)			28.448	<0.001
Yes	54 (50.5)	56 (22.1)		
No	53 (49.5)	197 (77.9)		
Mechanical ventilation before surgery (*n*, %)			2.806	0.094
Yes	14 (13.1)	19 (7.5)		
No	93 (86.9)	234 (92.5)		
Health insurance (*n*, %)			0.065	0.799
No	106 (99.1)	248 (98.0)		
Yes	1 (0.9)	5 (2.0)		
Year of admission (*n*, %)	2013 (2010, 2016)	2013 (2009, 2017)	−0.361	0.718
Gross classification (*n*, %)			11.385	0.003
A/B	7 (8.2)	6 (2.4)		
C/D	73 (85.9)	204 (80.6)		
E	5 (5.9)	43 (17.0)		
Associated anomalies (*n*, %)			22.506	<0.001
Yes	56 (76.7)	112 (45.2)		
No	17 (23.3)	136 (54.8)		
Duration of hospital stay (median, day)	1 (1, 2)	22 (16, 31)	−14.560	<0.001
Duration of stay in intensive care unit (median, day)	0 (0, 1)	7 (7, 19)	−8.267	<0.001
Hospitalization expense (median, CNY)	3,763.1 (2,168.2, 6,965.1)	42,673.1 (24,309.4, 65,877.5)	−14.472	<0.001

### Risk Factors and Reasons for Treatment Abandonment Before Surgery

In order to find the risk factors for treatment abandonment before surgery, we conducted a multivariate analysis. ROC curve analysis was used to determine the stratification value for age at diagnosis, as well as mother's gravidity and parity according to the maximum combined sensitivity and specificity values. The cutoff values for the above characteristics were 4 days, 1, and 1, respectively. As shown in Table 2, multivariate analysis showed that parity >1, unplanned admission to intensive care unit before surgery, associated anomalies, and Gross type A/B were significant independent risk factors for treatment abandonment before surgery.

**Table 2:** Multivariate logistic regression analysis of prediction of treatment abandonment before surgery.

**Variables**	**Estimate**	**Standard error**	**Wald**	***P*-value**	**OR (95% CI)**
Parity >1	1.108	0.330	11.252	0.001	3.029 (1.585, 5.789)
Unplanned admission to intensive care unit before surgery	1.494	0.332	20.250	<0.001	4.454 (2.324, 8.536)
Associated anomalies	1.040	0.346	9.059	0.003	2.830 (1.437, 5.572)
Gross type A/B	1.694	0.668	6.421	0.011	5.441 (1.468, 20.167)
Gross type E	−0.988	0.583	2.876	0.090	0.372 (0.119, 1.166)

According to the electronic medical records, the reasons for 107 patients who abandoned treatment before surgery included financial difficulties (*n* = 10), multiple malformations with poor prognosis (*n* = 9), belief in incurability and concerns about the prognosis of the diseases (*n* = 20), and unknown (*n* = 74).

### Comparison Between Treatment Abandonment After Surgery and Non-treatment Abandonment Groups

As shown in Table 3, by comparing clinical characteristics between treatment abandonment after surgery (*n* = 59) and non-treatment abandonment (*n* = 194) groups, we found significant differences in age at diagnosis, birth weight, department of discharge, year of admission, distance between the proximal and distal esophageal pouches, length of hospital stay, hospitalization expense, unplanned admission to intensive care unit after surgery, type of surgery, operative time, option to feed before discharge, parenteral nutrition treatment, extubation before discharge, pneumothorax, and anastomotic leakage (all *P* < 0.05). However, there were no differences in other clinical characteristics between the two groups (all *P* > 0.05).

**Table 3:** Comparison between treatment abandonment after surgery and non-treatment abandonment groups.

**Variables**	**Treatment abandonment after surgery (*n* = 59)**	**Non-treatment abandonment (*n* = 194)**	**Results**	***P*-value**
Gender (*n*, %)			0.575	0.448
Boy	40 (67.8)	121 (62.4)		
Girl	19 (32.2)	73 (37.6)		
Age at diagnosis (median, days)	2 (1, 3)	2 (1, 10)	−2.587	0.010
Birth weight (median, g)	2,805 ± 531	2,960 ± 477	−2.100	0.037
Gestational age (*n*, %)			0.345	0.557
Preterm	7 (12.1)	18 (9.4)		
Term/overdue	51 (87.9)	173 (90.6)		
Mode of delivery (*n*, %)			0.066	0.798
Cesarean delivery	37 (66.1)	122 (64.2)		
Spontaneous vaginal delivery	19 (33.9)	68 (35.8)		
Gravidity (median)	1 (1, 2)	1 (1, 2)	−0.188	0.851
Parity (median)	2 (1, 3)	2 (1, 2)	−0.699	0.484
Twin (*n*, %)			0.223	0.636
No	53 (91.4)	180 (94.2)		
Yes	5 (8.6)	11 (5.8)		
Geographic location (*n*, %)			0.693	0.405
City	28 (47.5)	103 (53.6)		
Village	31 (52.5)	89 (46.4)		
Father's occupation (*n*, %)			0.480	0.855
Office staff/vendor/worker	33 (69.2)	92 (69.2)		
Farmer	7 (15.6)	27 (20.3)		
Unemployed	5 (11.1)	14 (10.5)		
Mother's occupation (*n*, %)			1.242	0.537
Office staff/vendor/worker	18 (46.2)	70 (53.4)		
Farmer	7 (17.9)	26 (19.8)		
Unemployed	14 (35.9)	35 (26.7)		
Father's age (median, years)	28 (27, 33)	31 (27, 35)	−1.579	0.114
Mother's age (median, years)	28 (25, 28)	29.0 (26, 33)	−1.518	0.129
Siblings (*n*, %)			0.006	0.938
Yes	22 (37.9)	71 (37.4)		
No	36 (62.1)	119 (62.6)		
Elder brother (*n*, %)			0.473	0.491
Yes	8 (15.4)	20 (11.8)		
No	44 (84.6)	150 (88.2)		
Elder sister (*n*, %)			0.553	0.457
Yes	8 (15.4)	34 (20.0)		
No	44 (84.6)	136 (80.0)		
Urgency of admission (*n*, %)			1.206	0.272
Emergency	44 (74.6)	130 (67.0)		
Planned	15 (25.4)	64 (33.0)		
Department of admission (*n*, %)			1.578	0.448
Neonatal surgery	42 (71.2)	126 (64.9)		
Neonatal center/ Intensive care unit	15 (25.4)	52 (26.8)		
Others	2 (3.4)	16 (8.2)		
Department of discharge (*n*, %)			44.759	<0.001
Neonatal surgery	13 (22.0)	129 (66.5)		
Neonatal center/intensive care unit	44 (74.6)	50 (25.8)		
Others	2 (3.4)	15 (7.7)		
Unplanned admission to intensive care unit before surgery (*n*, %)			0.544	0.461
Yes	11 (18.6)	45 (23.2)		
No	48 (81.4)	149 (76.8)		
Mechanical ventilation before surgery (*n*, %)			0.276	0.600
Yes	3 (5.1)	16 (8.2)		
No	56 (94.9)	178 (91.8)		
Health insurance (*n*, %)			0.506	0.477
No	59 (100)	189 (97.4)		
Yes	0	5 (2.6)		
Year of admission (*n*, %)	2011 (2009, 2014)	2014 (20010, 2018)	−2.666	0.008
Gross classification (*n*, %)			2.562	0.109
C	50 (84.7)	145 (74.7)		
A/B/D/E	9 (15.3)	49 (25.3)		
Distance between the proximal and distal esophageal pouches (median, cm)	2.0 (1.0, 3.0)	1.5 (1.0, 2.2)	−2.528	0.011
Associated anomalies (*n*, %)			2.034	0.154
Yes	29 (53.7)	83 (42.8)		
No	25 (46.3)	111 (57.2)		
Duration of hospital stay (median, day)	11 (5, 20)	24 (18, 33)	−7.337	<0.001
Duration of stay in intensive care unit (median, day)	5 (1, 11)	8 (0, 21)	−1.404	0.160
Hospitalization expense (median, CNY)	31,081.1 (16,140.5, 43,454.2)	48,327.4 (25,840.1, 70,508.7)	−4.244	<0.001
Planned admission to intensive care unit after surgery (*n*, %)			1.099	0.294
Yes	24 (40.7)	94 (48.5)		
No	35 (59.3)	100 (51.5)		
Unplanned admission to intensive care unit after surgery (*n*, %)			38.631	<0.001
Yes	23 (39.0)	13 (6.7)		
No	36 (61.0)	181 (93.3)		
Type of surgery (*n*, %)			6.633	0.028
Thoracoscopy	9 (15.3)*^a^*	55 (28.4)		
Open surgery	46 (78.0)*^b^*	135 (69.6)*^c^*		
Converted	4 (6.8)*^a^*	4 (2.1)		
Operative time (median, min)	150.7 ± 54.6	127.5 ± 44.6	−3.076	0.008
Option to feed before discharge (*n*, %)			194.125	<0.001
Full oral	3 (5.2)	159 (82.4)		
Tube feeding	1 (1.7)	27 (14.0)		
Total parenteral nutrition	54 (93.1)	7 (3.6)		
Parenteral nutrition treatment (median, days)	8 (4, 17)	16 (11, 23)	−8.543	<0.001
Duration of mechanical ventilation (median, day)	50 (0, 191)	43 (0, 142)	−1.305	0.192
Extubation before discharge (*n*, %)			6.216	0.013
Yes	55 (93.2)	193 (99.5)		
No	4 (6.8)	1 (0.5)		
Pneumothorax (*n*, %)			35.888	<0.001
Yes	36 (72.0)	51 (26.4)		
No	14 (28.0)	142 (73.6)		
Anastomotic leakage (*n*, %)			18.317	<0.001
Yes	29 (61.7)	55 (28.5)		
No	18 (38.3)	138 (71.5)		

a *Two patients only underwent exploratory surgery*.

b *One patient only underwent exploratory surgery and two patients only underwent gastrostomy*.

c *One patient underwent staged gastrostomy and died of pneumonia 1 month after operation*.

### Risk Factors and Reasons for Treatment Abandonment After Surgery

In order to find the risk factors for treatment abandonment after surgery, we conducted a multivariate analysis. ROC curve analysis was used to determine the stratification value for age at diagnosis, birth weight, year of admission, distance, and operative time according to the maximum combined sensitivity and specificity values. The cutoff values for the above characteristics were 4 days, 2,545 g, 2016, 2.9 cm, and 133 min, respectively. As shown in Table 4, multivariate analysis showed that birth weight <2,545 g, discharged from neonatal center/intensive care unit and other departments, unplanned admission to intensive care unit after surgery, operative time >133 min, admission before 2016, pneumothorax, and anastomotic leakage were significant independent risk factors for treatment abandonment after surgery.

**Table 4:** Multivariate logistic regression analysis of prediction of treatment abandonment after surgery.

**Variables**	**Estimate**	**Standard error**	**Wald**	***P*-value**	**OR (95% CI)**
Birth weight >2,545 g	−1.649	0.605	7.418	0.006	0.192 (0.059, 0.630)
Discharged from neonatal center/intensive care unit	2.465	0.547	27.370	<0.001	17.491 (5.987, 51.101)
Discharged from other departments	3.152	1.063	8.796	0.003	23.392 (2.913, 187.855)
Unplanned admission to intensive care unit after surgery	2.135	0.573	13.898	<0.001	8.459 (2.753, 25.992)
Operative time > 133 min	1.158	0.520	4.954	0.026	3.183 (1.148, 8.823)
Year of admission >2016	−1.147	0.700	2.685	0.101	0.318 (0.081, 1.252)
Pneumothorax	1.790	0.552	10.494	0.001	5.987 (2.028, 17.676)
Anastomotic leakage	1.098	0.536	4.197	0.041	2.999 (1.049, 8.576)

According to the electronic medical records, the reasons for 59 patients who abandoned treatment after surgery included postoperative complications (*n* = 50), financial difficulties (*n* = 2), excessive length of intensive care unit stay (*n* = 2), belief in incurability and concerns about the prognosis of the diseases (*n* = 7), multiple malformations with poor prognosis (*n* = 2), and unknown (*n* = 1).

## Discussion

To our knowledge, this is the first study to explore risk factors and reasons for treatment abandonment of EA/TEF patients. This study showed that patients in critical condition, associated anomalies, Gross type A/B, and occurrence of complications were high-risk groups for treatment abandonment. Possible reasons included financial embarrassment, multisystem malformations, parents' belief in incurability of the disease or anxieties toward poor outcome, and extensive length of intensive care unit stay.

A cross-sectional study concluded that age, gender, and geography location were the predictors of patients discharge against medical advice (DAMA) (6). A recent study from Australia found that predictors of DAMA were hospital site, a mental health/behavioral diagnosis, aboriginality, emergent rather than elective admissions, a gastrointestinal diagnosis, and a history of previous DAMA (7). A study associated with treatment abandonment of children with malignant solid tumors in Peru showed that rural origin and lack of formal parental employment were independently predictive of treatment abandonment (8). However, our findings were inconsistent with the above results. Possible explanations could be that the situation in China is different, and that EA/TEF, a congenital malformation of neonates, has completely different characteristics from the diseases reported in previous studies.

One of the most crucial reasons for treatment abandonment is financial embarrassment. China has developed a health reform plan which expanded insurance coverage to about 90% of the population and established a national essential medicine program. As a result, primary care services have improved and patients in both urban and rural areas all have access to basic healthcare. However, most newborns have no health insurance, so families in poverty cannot afford to pay for the treatment fee of these babies. When reviewing the medical records in this study, only a few parents mentioned that the reasons for treatment abandonment included financial difficulties, but in reality, it is a burden that continues to preside clinically. However, we were unable to obtain relevant information due to retrospective data collection and the associated difficulties in interviewing these parents. In addition, the information (geographic location, parents' occupation, health insurance) that we included in the analysis of the risk factors for treatment abandonment was also closely related to family economic condition, but these factors did not show statistical significance between the two groups. Therefore, in future clinical work and research, more attention should be paid to income and education levels of the parents as well as other economic and social factors, as they are crucial for understanding the causes of treatment abandonment.

Another reason could be that in our country, most parents barely have any medical knowledge, and their understanding about the treatment, complications, and prognosis of the disease largely depends on the doctor's explanation and advice. Doctors play a very important role in helping parents understand the disease and subsequent medical choices. However, doctors from the emergency department and intensive care unit tend to receive patients who are in critical condition and thus are more likely to emphasize on poor prognosis and the high treatment cost. In addition to the severity of the patients' disease, this also partly explained the higher proportion of treatment abandonment in patients who had been admitted into the intensive care unit. In order to minimize the subjective factors arising from various levels of EA/TEF recognition in doctors, we have formed a disease-explaining model for clinical use. Explanation of the disease is now a multidisciplinary discussion including surgical, emergency, and neonatal departments, aimed to provide parents with a more rounded and balanced understanding.

In China, the success rate of EA/TEF has generally improved. In particular, the survival rate of type C EA/TEF without severe malformation reported in the relevant literature is higher than 90% (9). Nonetheless, this disease still has many postoperative complications, including anastomotic stricture (9–80%), anastomotic leakage (5–20%), and recurrent TEF (5–10%) (9–26). In this study, we also found that severe postoperative complications and excessive length of intensive care unit stay were vital reasons leading to abandoning treatment after surgery. According to our long-term follow-up and observation, prevention and treatment of postoperative complications are the key to prevent poor long-term outcome and avoid treatment abandonment after surgery. As shown in Supplementary Figure 1, since 2016, there has been a significant change in the proportion of abandonment after surgery, from 20.31 to 6.73%. Between 2015 and 2016, with the progress of surgical technology and nursing methods, standardized and multidisciplinary EA/TEF management, close cooperation and communication between the intensive care unit department and parents, the improvement of parents' economic and education level, and improvement of medical insurance system, there has been great progress in the treatment of EA/TEF which has resulted in the sharp decline of abandonment after surgery.

There is no report on the topic of treatment abandonment for EA/TEF, but treatment refusal and abandonment continue to be big challenges in China, as demonstrated from domestic research on abandonment of treatment. Based on the above analysis, we suggest the following clinical actions to avoid abandonment of treatment as much as possible. First, parents and physicians should regularly communicate with each other about the overall assessment of the patient's condition, the family's expectation, and the physician's experiences. Inaccurate or missing information may skew their understanding and affect their decisions. Second, timely and systematic health education should be carried out to avoid treatment abandonment due to parents' misunderstanding of the disease. EA/TEF is not incurable. Third, improvement of surgical technology and nursing methods, as well as prevention and treatment of postoperative complications, are the key to prevent poor prognosis and avoid treatment abandonment after surgery. Although very common, attentive care and active treatment of postoperative complications have significantly increased the success rate. Finally, although medical insurance system has improved significantly, China is still a developing country, and most cities are still in the low-income level. Thus, we should provide poor parents with funding, or help with online fund-raising and other such means to help them out of the financial difficulties.

One of the limitations of the study is that our single-center retrospective study cannot fully represent the overall situation of China. Although abandonment of treatment is faced with ethical, socioeconomic, and legal issues, it is still an important topic worthy of in-depth study. The other limitations include a lack of detailed records for the reasons for abandonment, and no follow-up investigations for the patients who abandoned treatment. Due to the limitations of this study, we will conduct qualitative studies to further explore the reasons for treatment abandonment.

In conclusion, this study provides us with a better understanding of the risk factors and reasons for treatment abandonment for patients with EA/TEF. With the conclusions we have arrived at through this analysis, we aim to further improve the level of clinical diagnosis and treatment, apply for subsidies for families with financial difficulties, spread relevant knowledge of EA/TEF to primary hospitals, maternity hospitals, and the public, and formulate management policies to prevent abandonment of treatment and protect these children in the future.

## Data Availability Statement

The raw data supporting the conclusions of this article will be made available by the authors, without undue reservation.

## Ethics Statement

The studies involving human participants were reviewed and approved by the Medical Ethics Committee of Beijing Children's Hospital (2019-k-333). Written informed consent from the participants' legal guardian/next of kin was not required to participate in this study in accordance with the national legislation and the institutional requirements.

## Author Contributions

JH and SY: study conception and design. SiL, PW, and YZhao: data acquisition. KH, YZhan, YG, and ShL: analysis and data interpretation. SY and JL: drafting of the manuscript. All authors contributed to the article and approved the submitted version.

## Conflict of Interest

The authors declare that the research was conducted in the absence of any commercial or financial relationships that could be construed as a potential conflict of interest.

## Supplementary Material

Supplementary Figure 1 The proportion of treatment abandonment and non-treatment abandonment in different years.
