# Supplementary figures and images for: Risk Factors and Reasons for Treatment Abandonment for Patients With Esophageal Atresia: A Study From a Tertiary Care Hospital in Beijing, China
Source: Front Pediatr. 2021 Apr 27;9:634573. doi: 10.3389/fped.2021.634573 (PMC8112547; doi:10.3389/fped.2021.634573)

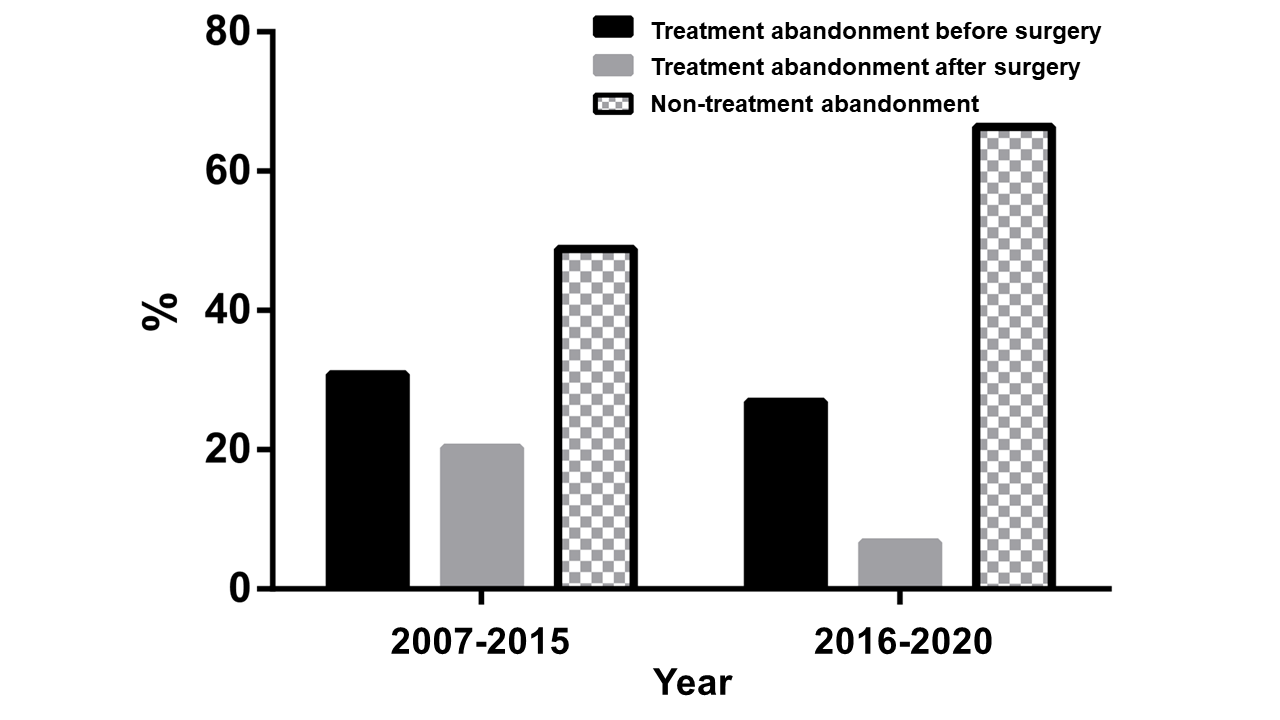

Supplement: Supplementary Figure 1 — The proportion of treatment abandonment and non-treatment abandonment in different years. [file Image_1.TIF]
